# Synergistic, long-term effects of glutamate dehydrogenase 1 deficiency and mild stress on cognitive function and mPFC gene and miRNA expression

**DOI:** 10.1038/s41398-023-02534-y

**Published:** 2023-07-07

**Authors:** Kfir Asraf, Hiba Zaidan, Baylasan Natoor, Inna Gaisler-Salomon

**Affiliations:** 1 School of Psychological Sciences, Department of Psychology, University of Haifa, Haifa, 3498838 Israel; 2 The Integrated Brain and Behavior Research Center (IBBRC), University of Haifa, Haifa, 3498838 Israel

**Keywords:** Molecular neuroscience, Learning and memory, Epigenetics and behaviour, Schizophrenia

## Abstract

Glutamate abnormalities in the medial prefrontal cortex (mPFC) are associated with cognitive deficits. We previously showed that homozygous deletion of CNS glutamate dehydrogenase 1 (*Glud1*), a metabolic enzyme critical for glutamate metabolism, leads to schizophrenia-like behavioral abnormalities and increased mPFC glutamate; mice heterozygous for CNS *Glud1* deletion (C-*Glud1*^+/−^ mice) showed no cognitive or molecular abnormalities. Here, we examined the protracted behavioral and molecular effects of mild injection stress on C-*Glud1*^+/−^ mice. We found spatial and reversal learning deficits, as well as large-scale mPFC transcriptional changes in pathways associated with glutamate and GABA signaling, in stress-exposed C-*Glud1*^+/−^ mice, but not in their stress-naïve or C-*Glud1*^+/+^ littermates. These effects were observed several weeks following stress exposure, and the expression levels of specific glutamatergic and GABAergic genes differentiated between high and low reversal learning performance. An increase in miR203-5p expression immediately following stress may provide a translational regulatory mechanism to account for the delayed effect of stress exposure on cognitive function. Our findings show that chronic glutamate abnormalities interact with acute stress to induce cognitive deficits, and resonate with gene x environment theories of schizophrenia. Stress-exposed C-*Glud1*^+/−^ mice may model a schizophrenia high-risk population, which is uniquely sensitive to stress-related ‘trigger’ events.

Intact glutamate transmission in the prefrontal cortex (PFC) is critical for spatial learning, cognitive flexibility, social recognition and problem solving [1–6], and is disrupted in several psychiatric disorders including schizophrenia [7, 8]. While most research pertaining to glutamate disruption and cognitive dysfunction in schizophrenia has focused on the NMDA receptor [9–11], recent attention has turned to manipulations that target glutamate synthesis, release or reuptake. Indeed, such manipulations significantly impact learning, memory and attention [12–14].

Glutamate Dehydrogenase 1 (GLUD1 in humans, GDH in rodents), encoded by the *Glud1* gene, is a key regulator of glutamate metabolism in CNS. GDH is a mitochondrial enzyme, expressed mainly in astrocytes [15], where it catabolizes glutamate to α-ketoglutarate [16]. Our previous studies in mice with a homozygotic deletion of *Glud1* in CNS (C-*Glud1*^−/−^ mice) revealed elevated hippocampal cerebral blood volume (CBV) and glutamate levels in medial PFC (mPFC) and hippocampus, along with baseline and amphetamine-induced hyperlocomotion. These findings resonate with human schizophrenia data showing decreased *Glud1* expression [14], elevated hippocampal CBV and glutamate/glutamine (Glx) levels [17, 18] and a hyperdopaminergic response to amphetamine [19]. C-*Glud1*^−/−^ mice also show prominent abnormalities in tasks relevant to cognitive impairment in schizophrenia, including working, spatial and social memory tasks as well as reversal and extra-dimensional set shifting (EDSS) in the Water T-maze [14, 20]. Unlike C-*Glud1*^−/−^ mice, heterozygous C-*Glud1*^+/−^ mice show no substantial changes in glutamate levels in hippocampus or PFC, and control-like behavior in a range of cognitive tasks with the exception of the challenging EDSS task [14].

Stress exposure in late adolescence or early adulthood may trigger symptom eruption in schizophrenia and related disorders. Animal studies have shown that stress exposure hinders PFC-dependent working memory, social recognition and cognitive flexibility [21, 22]. Impaired glutamate signaling is implicated in stress-induced cognitive deficits [23, 24]. Furthermore, in support of an interaction between genetic and environmental factors, stress exposure alters the behavioral consequences of genetic manipulations affecting the excitatory/inhibitory (E/I) balance in tasks measuring cognitive flexibility and other PFC-dependent behaviors [25, 26]. Notably, acute stress was found to increase NMDAR- and AMPAR-mediated synaptic currents in PFC pyramidal neurons [27, 28], while chronic stress attenuates acute stress-evoked increases in extracellular glutamate [29].

Preliminary data from our lab [30] indicate that social isolation stress renders heterozygote C-*Glud1*^+/−^ mice susceptible to deficits in mPFC-dependent reversal learning [31], which are absent in social stress-naïve mice. However, we and others have shown that exposure to social isolation stress alone leads to a wide range of behavioral deficits, including spatial acquisition and reversal abnormalities [32–34]. Chronic social isolation stress exposure also leads to abnormalities in glutamate homeostasis [34–36]. The impact of acute stress, in particular mild stress with negligible consequences in genetically intact mice, was not previously examined in C-*Glud1*^+/−^ mutants.

In the present study, we aimed to parse out the impact of stress and *Glud1* deficiency using a mild acute stress protocol [37]. We first asked whether acute stress exposure in early adulthood would ‘trigger’ cognitive abnormalities in C-*Glud1*^+/−^ mice. We hypothesized that heterozygous C-*Glud1*^+/−^ mice exposed to stress would exhibit deficits in capacities that require intact hippocampal and mPFC function, i.e., spatial rule acquisition, cognitive flexibility and social function, whereas *Glud1* heterozygosity or stress exposure alone would not elicit behavioral abnormalities.

Second, we aimed to recognize unique gene expression patterns in the mPFC of acute stress-exposed CNS-*Glud1*^+/−^ mice, using genome-wide RNA sequencing (RNA-seq). We hypothesized that acute mild stress would have little effect on its own, but when combined with *Glud1* deficiency would induce long-term changes in mPFC gene expression patterns. In light of previous studies on the separate effects of *Glud1* deficiency and stress [14, 20], we hypothesized that glutamate and GABA signaling pathways would be particularly affected in *Glud1-*deficient, stress-exposed mice.

Since behavioral and transcriptional changes were detected several weeks following stress exposure, we aimed to identify immediate stress-induced changes in mPFC. We examined immediate and long-term changes in the expression of several microRNA (miRNA) molecules, which play a key role in translational regulation [38] and were shown to be associated with stress and cognition [39–45]. We hypothesized that changes in the expression of these miRNAs would occur immediately following stress exposure and would persist to adulthood in stress-exposed C-*Glud1*^+/−^ mice but not in their C-Cre+ or stress-naïve littermates.

## Results

### Behavior

**Fig. 1:**
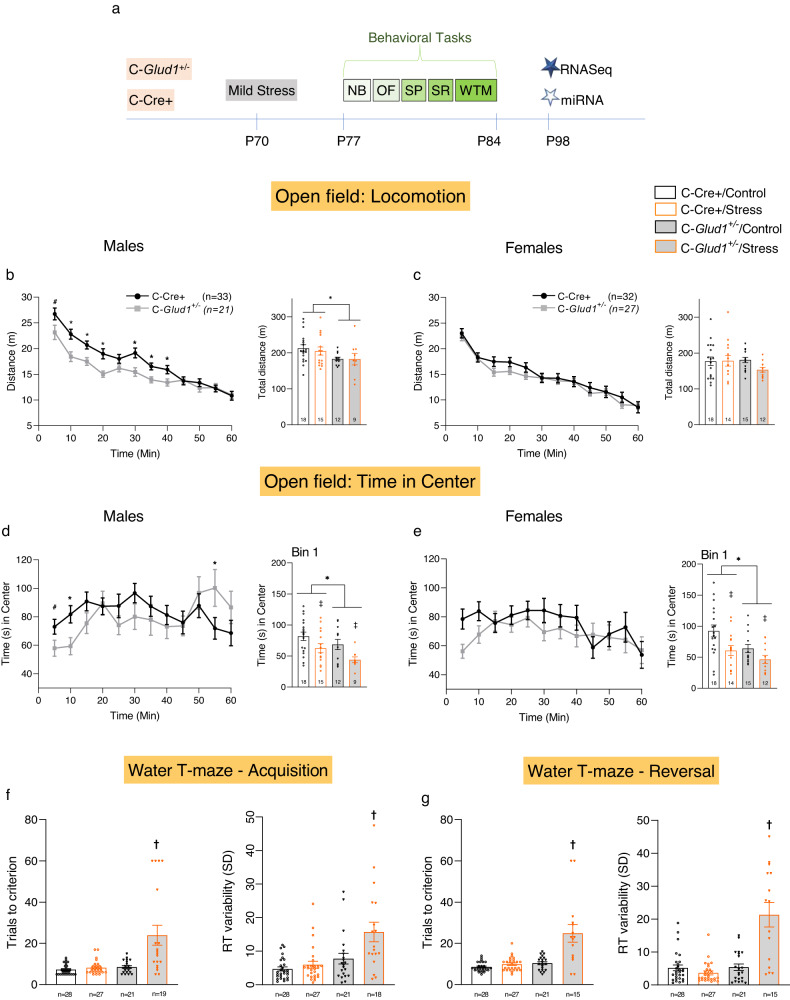
Stress exposure leads to cognitive deficits in C-*Glud1*^+/−^ mice. Timeline of the experiment (**a**); NB—Nest building, OF—Open field, SP—social preference, SR—social recognition, WTM—Water T-maze. In the Open Field, male C-*Glud1*^+/−^ mice traveled less distance during the 60-min test (**b**, left; Time*Genotype: *F*_(4.67,233.60)_ = 3.46, *p* = 0.005, η²_p_ = 0.064). Female mice exhibited no genotypic differences (**c**, left). Male C-*Glud1*^+/−^ mice exhibited an erratic pattern of time spent in the center of the open field (**d**, left; Time*Genotype: *F*_(6.16,308.46)_ = 2.68, *p* = 0.013, η²_p_ = 0.051). During the first 5 min (Bin 1), genotype and stress affected the time spent in the center (**d**, right; C-*Glud1*^+/−^<C-Cre+, Genotype: *F*_(1,50)_ = 4.49, *p* = 0.038, η²_*p*_ = 0.082; Stress<Control; Group: *F*_(1,50)_ = 8.47, *p* = 0.005, η²_p_ = 0.144). Female mice exhibited no genotypic or stress-induced differences across all bins (**e**, left). In Bin 1, C-*Glud1*^+/−^ and stress-exposed mice spent less time in the center (**e**, right; Genotype: F_(1,55) _= 6.64, *p* = 0.012, η²_p_ = 0.107; Grou*p*: F_(1,55)_ = 8.96, *p* = 0.004, η²_p_ = 0.140). In the Water T-maze, male and female data were combined. In the Acquisition phase, C-*Glud1*^+/−^/Stress mice required a larger number of trials to criterion (**f**, left; Genotype*Group: *F*_(1,90)_ = 12.68, *p* < 0.001, η²_p_ = 0.122) and exhibited higher RTv (**f**, right; Genotype*Group: *F*_(1,90)_ = 4.94, *p* = 0.029, η²_p_ = 0.052) compared to the other groups. The same pattern was observed in the Reversal phase (**g**, left, trials to criterion: Genotype*Group: *F*_(1,87)_ = 17.79, *p* < 0.001, η²_p_ = 0.170; **g**, right, RTv: Genotype*Group: *F*_(1,87)_ = 34.05, *p* < 0.001, η²_p_ = 0.281). ^#^*p* < 0.055, *p < 0.05 **p < 0.01 ***p < 0.001. ^‡^Main effect for stress. ^†^Group mean is statistically different from all others. NB, SP, and SR results were not significant and are provided in the SI.

Behavioral tests were run in the following order: Nesting Behavior (NB), Open Field (OF), Social Preference (SP), Social Recognition (SR) and Water T-Maze (WTM; Fig. 1a). Detailed statistical analysis is presented in Table S1.

In the NB test, all mice exhibited similar nesting behavior, with relatively high nesting scores regardless of Genotype, Group or Sex. No main effects or interactions were found (Fig. S1).

In the OF, males and females differed in total locomotor activity (Sex main effect; males>females) and in the time spent in center over time (Time*Sex interaction). Therefore, male and female data were analyzed separately. In male mice, all groups exhibited less locomotor activity over time, and C-*Glud1*^+/−^ mice traveled less distance compared to C-Cre+ controls, regardless of stress exposure, during the first 40 min (Fig. 1b_Left_) and over the entire test period (**1b**_**Right**_). No Group effect nor a Genotype*Group interaction was found. In female mice, all groups exhibited less locomotor activity over time (**1c**_**Left**_). We found no effect for Genotype, Group or a Genotype*Group interaction (**1c**_**Right**_).

Examination of time spent in the center of the OF in males revealed a main effect of Time and a Time*Genotype interaction. We found a significant change over time for C-*Glud1*^+/−^ mice, which exhibited linear and polynomial trends, indicative of ‘erratic’ behavior (Fig. 1b_Left_). There was no change over time in C-Cre+ mice. During the first 5 min of the test (bin 1, **1d**_**Right**_), C-*Glud1*^+/−^ mice spent less time in the center than their C-Cre+ controls, and stress-exposed mice spent less time in the center than their stress-naïve controls. No Genotype*Group interaction was found. In females, we found a main effect for Time, but no Time*Genotype interaction (**1e**_**Left**_). Similarly to male mice, C-*Glud1*^+/−^ females spent less time in the center compared to their C-Cre+ littermates, and stress exposure similarly led to a decrease in time in the center (**1e**_**Right**_). No Genotype*Group interaction was found.

In the social behavior tests, we found no Genotype, Group or Sex effects, nor interactions between them, in the SP or SR tasks (Fig. S1).

For the WTM test, male and female data were combined since no Sex effects or interactions were found. In the acquisition phase, we found Genotype*Group interactions for both Trials to Criterion and Reaction Time variability (RTv). Post-hoc tests for both measures indicate that C-*Glud1*^+/−^/Stress mice required more trials to criterion (Fig. 1f_Left_) and displayed higher RTv (**1f**_**right**_**;** Fig S2) than mice in all other groups. Data from 1 C-*Glud1*^+/−^/Stress mouse was excluded (>2 SD above mean) in the RTv analysis. RT data followed the same pattern of results: RTs were highest in the C-*Glud1*^+/−^/Stress group (not shown, see Table S1). Four mice from the C-*Glud1*^+/−^/Stress group [21.05%; significantly more than in other groups (0%)] failed to achieve criterion in the acquisition phase and received a maximal score of 60. These mice did not continue to the probe or reversal phase. In the reversal phase we found a Genotype*Group interaction for both Trials to Criterion and RTv. Post-hoc tests showed that C-*Glud1*^+/−^/Stress mice required more trials to criterion (**1g**_**Left**_) and displayed higher RTv (**1g**_**Right**_) than mice in all other groups. Higher RTs were observed in this group as well.

### RNA-seq

*Differentially expressed genes (DEGs)*—as can be seen in Table 1, RNA-seq analysis showed that 3107 genes (19.86% of the mPFC-expressed protein-coding genes) in the C-*Glud1*^+/−^/Stress group, 64 in the C-*Glud1*^+/−^/Control group, and 3 in the C-Cre+/Stress group were differentially expressed compared to C-Cre+/Control (FDR-corrected *q* value < 0.05). For a full list of DEGs in each comparison, see Table S2.

**Table 1:** Summary of protein-coding genes detected, tested and differentially expressed in the 3 experimental conditions.

Comparison (vs C-Cre+/Control)	Total number of detected genes (protein coding only)	Tested	Significantly upregulated	Significantly downregulated
C-Cre + /Stress	20,253	15,708	0	3
C-*Glud1*^+/−^/Control	20,253	15,705	7	57
C-*Glud1*^+/−^/Stress	20,253	15,640	1825	1282

Tested = genes analyzed for differential expression (i.e., had normalized counts above 10 for at least one sample in each group).

**Fig. 2:**
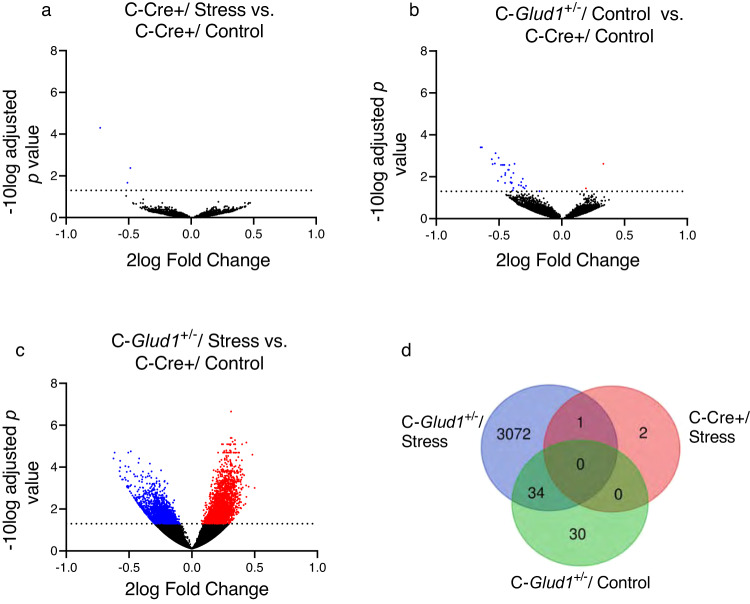
Extensive gene expression changes are uniquely found in mPFC of *C-Glud1*^+/−^ /Stress mice. Volcano plots demonstrating upregulated (red) and downregulated (blue) genes in C-Cre+/Stress (**a**), C-*Glud1*^+/−^/Control (**b**) and C-*Glud1*^+/−^/Stress (**c**) compared to C-Cre+/Control mPFC samples. Dotted lines indicate threshold for statistical significance (−10log > 1.3, corresponding to *q* < 0.05). The largest number of DEGs is found in C-*Glud1*^+/−^/Stress samples, mostly (~58%) upregulated. **d** Venn diagram of DEG overlap between conditions. 98.8% of DEGs were unique to the C-*Glud1*^+/−^/Stress group.

As can be seen in Fig. 2, in the C-Cre+/Stress group all 4 DEGs were downregulated (**2a**), in the C-*Glud1*^+/−^/Stress group the majority of the 64 DEGs were downregulated (**2b**), and the *Glud1*^+/−^/Stress group exhibited the highest number of DEGs, with 58.73% upregulated DEGs (**2c**). Examining the overlap between DEGs in our groups (**2d**), we found that 1 DEG in the C-Cre+/Stress group and 80% of the DEGs in the *Glud1*^+/−^/Control groups were also downregulated in the *Glud1*^+/−^/Stress group. There was only a small overlap between the C-*Glud1*^+/−^/Stress group and other groups: out of 3,107 DEGs, 3,072 (98.87%) were unique to the C-*Glud1*^+/−^/Stress group.

**Fig. 3:**
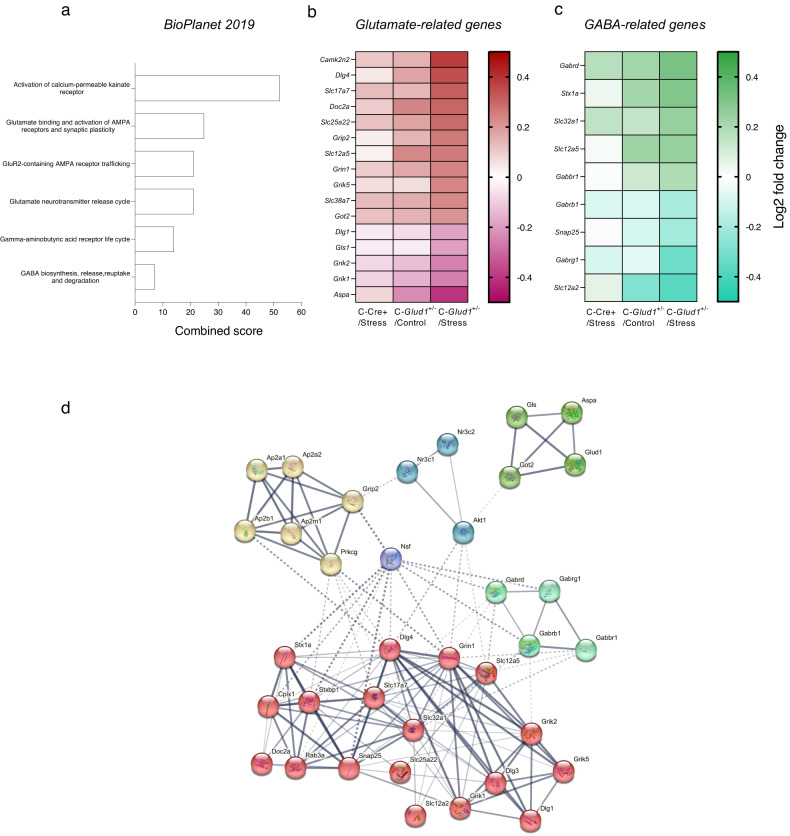
Destabilized Glutamatergic and GABAergic Homeostasis in Stress-Exposed C-*Glud1*^+/−^ Mice. **a** Top enriched BioPlanet 2019 terms for DEGs in C-*Glud1*^+/−^/Stress mice include glutamate- and GABA-related pathways. Combined score is computed as log(*p* value)*Z score. **b**, **c** Relative expression of glutamatergic and GABAergic genes in C-Cre+/Stress (left; *n* = 7), C-*Glud1*^+/−^/Control (middle; *n* = 5) and C-*Glud1*^+/−^/Stress groups (right; *n* = 9), relative to the C-Cre+/Control reference group (*n* = 7). Significant expression changes in both pathways were found in the C-*Glud1*^+/−^/Stress group alone. **d** Functional diagram of protein-protein interactions of glutamatergic, GABAergic and stress-related DEGs in C-Glud1^+/−^/Stress mice, compared to C-Cre+/Control mice. Line thickness indicates interaction strength. Dotted lines: interactions between proteins from different clusters.

To detect enriched biological categories, we performed an over-representation analysis (ORA) of unique DEGs in the C-*Glud1*^+/−^/Stress group using Enrichr [46, 47]. As can be seen in Fig. 3a, a BioPlanet 2019 ORA revealed significant changes in glutamate- and GABA-related pathways. These pathways were also prominent when other gene databases were used (e.g., GO Biological Process, Elsevier Pathway Collection; Fig S3a, b). The Elsevier Pathway Collection also pointed to the corticotropin-releasing hormone (CRH) secretion regulation pathway. Top terms also included S6K1 signaling and downregulation of ERBB4 signaling (BioPlanet), as well as chromatin organization involved in negative regulation of transcription, histone arginine methylation and postsynaptic density protein 95 clustering (GO Biological). For a list of the top 30 terms in each of the databases, see Table S3.

We then proceeded to examine expression patterns of functionally significant genes that contribute to homeostasis at the glutamate tripartite synapse, the E/I balance and cognitive function [8, 48]. C-*Glud1*^+/−^/Stress mice consistently showed expression changes in glutamate- and GABA-related genes (Fig. 3b, c). Interestingly, while *Glud1* expression was not significantly altered, many of the genes that contribute to glutamate and GABA homeostasis were differentially expressed in the C-*Glud1*^+/−^/Stress group, but not in C-Cre+/Stress or C-*Glud1*^+/−^/Control mice (Fig S3a, b; see Table S4a, b for DEGs from select over-represented categories). The HPA axis function-associated genes *Nr3c1* and *Nr3c2* were also differentially expressed exclusively in this group.

We examined in-silico the cell-type enrichment of the DEGs in C-*Glud1*^+/−^/Stress mice using the PanglaoDB Augmented 2021 database [49] (Enrichr), and found that neurons [Combined score (CS) = 55.62], particularly pyramidal ones (CS = 22.20), were enriched. GABAergic neurons were also enriched (CS = 19.63), as were oligodendrocytes (CS = 11.09), but astrocytes were not (CS = 2.92). No cell-specific enrichment was detected in C-*Glud1*^+/−^/Control mice.

The STRING database [50] was used to examine protein-protein interactions between glutamatergic, GABAergic and stress-related DEGs (Fig. S4). This analysis revealed a functional network with 6 clusters: Glutamatergic signaling (Red; 18 genes), Postsynaptic neurotransmitter receptor internalization (Yellow; 6 genes), Glutamate biosynthetic process (Dark green; 4 genes), GABAergic signaling pathway (Pale green; 4 genes), Stress (Blue; 3 genes) and *Nsf* (Purple; one gene). As can be seen in Fig. 3e, proteins encoded by DEGs interact within and between clusters.

We also examined whether genes known to interact with *Glud1* (STRING) were differentially expressed in C-*Glud1*^+/−^/Stress mice. Out of 63 genes known to interact with *Glud1*, 16 were differentially expressed in C-*Glud1*^+/−^/Stress mice (11 upregulated). Protein-protein interactions between these genes are shown in Fig. S5.

**Fig. 4:**
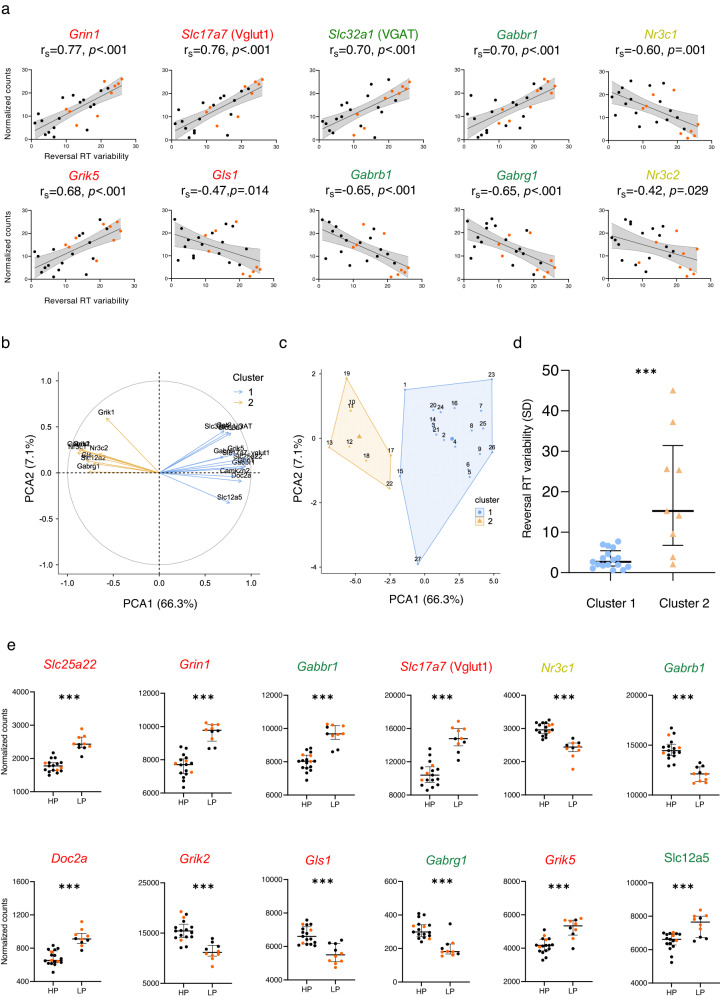
Reversal learning is associated with DEG expression levels. **a** RTv in the Water T-maze Reversal task correlates with expression levels of glutamatergic, GABAergic and stress-related DEGs. **b** K-means cluster analysis of these DEGs produces two distinct clusters. **c** Samples are clearly distributed between the two clusters (dots/triangles: mouse numbers). **d** Cluster 2 Reversal RTv scores are significantly higher than Cluster 1 scores (*Z* = −3.36, *p* < 0.001). **e** Glutamate, GABA and stress-related DEGs (presented in order of cluster association strength) are differentially expressed in HP (Cluster 1) and LP (Cluster 2) groups (all Z’s>3.31, FDR-corrected *p*’s < 0.01). ***p < 0.001.

To assess the relationship between mPFC gene expression and behavior, we calculated Spearman correlations between DEG transcript levels and behavioral measures in the open field and T-maze tasks, in all mice. Thirty-eight glutamatergic, GABAergic and stress-related genes differentially expressed exclusively in the C-*Glud1*^+/−^/Stress group (see Fig. 3b, c, S4) were selected. As can be seen in Table S4, transcript levels correlated with several behavioral measures, but mostly with individual RTv in the Water T-Maze Reversal task. RTv values predicted transcript levels of 33 out of the 38 genes. Figure 4a depicts select correlations between glutamatergic, GABAergic and stress-related DEGs and individual RTv (higher RTv reflects compromised cognitive performance), derived from all 4 groups.

K-means cluster analysis on these genes (jamovi (v2.2.5) ‘snowCluster’ module (v6.7.0)), excluding 2 genes due to low factor loading (Standardized;<|0.40|) with either cluster), yielded two clusters (Fig. 4b). The final model (Table S5) accounted for 74.3% of the variance, with the first PCA accounting for 66.33% of the variance. The 2 clusters separated the samples into two non-overlapping groups (**4c**), which differed in expression levels of clustered genes (samples in the ‘blue’ cluster have high expression levels of genes marked in blue and low expression levels genes marked in orange, and vice versa). Notably, these clusters differ in Reversal RTv scores (Mann–Whitney test; *Z* = −3.36, *p* < 0.001; **4d**, Table S5). High- and low- performance mice (HP and LP, respectively) displayed differential expression of all genes in the analysis; the top 12 differentiating genes are presented in **4e** (Mann–Whitney tests; all Zs>3.31, all *p’*s < 0.001). Notably, the majority of LP/Cluster 2 mice were C-*Glud1*^+/−^/Stress mice (orange dots; 60% of cluster 2; 33.33% of total sample).

### miRNA expression

**Fig. 5:**
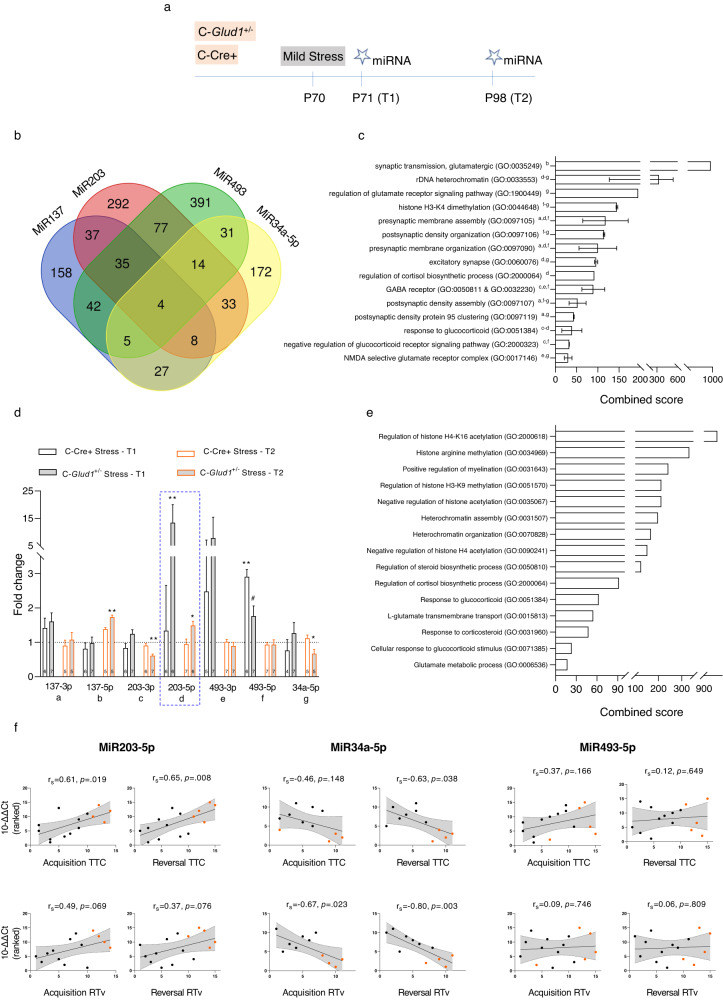
Specific miRNAs are differentially expressed in C-*Glud1*^+/−^/Stress mPFC and are associated with cognitive performance. **a** Timeline of miRNA experiment. **b** Venn diagram of target overlap between selected miRNAs. **c** MiRNA target shared pathways. Pathways shared by at least 2 miRNAs are presented. ^a^ MiR137-3p, ^b^ MiR137-5p, ^c^ MiR203-3p, ^d^ MiR203-5p, ^e^ MiR493-3p, ^f^ MiR493-5p, ^g^ MiR34a-5p. The combined score is calculated as the mean of the relevant miRNA target combined scores. **d** At T1 (immediately after stress), the expression of miR203-5p was elevated in mPFC of C-*Glud1*^+/−^ /Stress mice (*H*_(2)_ = 9.73, *p* = 0.002), and miR493-5p was upregulated in both C-*Glud1*^+/−^/Stress and C-Cre+/Stress groups (*H*_(2)_ = 8.46, *p* = 0.008), relative to C-Cre+/Control mice. At T2 (one month after stress), miR203-5p and miR137-5p were upregulated (*H*_(2)_ = 6.89, *p* = .024 and *H*_(2)_ = 10.05, *p* < 0.001, respectively) whereas miR203-3p and miR34a-5p were downregulated (*H*_(2)_ = 10.59, *p* = 0.001 and *H*_(2)_ = 9.64, *p* = 0.001, res*p*ectively). Post hocs: #*p* < 0.075, *p < 0.05, **p < 0.01. C-Cre+/Control group sample size: T1 (*n* = 5-6), T2 (*n* = 4–5). **e** Pathway analysis of transcriptional targets of miR203-5p, which was upregulated at both T1 and T2. **f** Expression levels of miR203-5p and miR34a-5p, but not miR493-5p, correlate with performance in acquisition and/or reversal of the Water T-maze. Orange dots depict C-*Glud1*^+/−^/Stress mice.

The timeline of the experiment is presented in Fig. 5a. MiRNA molecules were selected according to their relevance to stress regulation [42, 44] and cognitive function [40, 43]. For example, increased miR203 expression was found in human epileptic brains and in a mouse model of the disease, suggesting it is involved in regulating the E/I balance [41]. Increased miR203 was also induces microglial activation and production of pro-inflammatory cytokines in mouse hippocampus, as well as impaired learning [45]. We first conducted an in silico search (using the DIANA miRNA database) for targets of our selected miRNA molecules (shown in the literature to be affected by stress and associated with cognitive function) which overlap with DEGs that emerged from the RNA-seq data. We found an approximate average of 18% (SD = 0.01%) overlap between DEGs and targets of each miRNA. As can be seen in Fig. 5b, several of miRNA targets, also differentially expressed in C-*Glud1*^+/−^/Stress mice, were shared by the selected miRNAs. The miRNAs examined were found to target genes related to glutamate, GABA and postsynaptic components in pathway analysis conducted with GO biological, cellular and molecular databases; several miRNAs were found to affect the same pathways (**5c**).

Next, we asked whether the expression of these miRNAs is altered in the mPFC of C-*Glud1*^+/−^/Stress mice. Detailed statistical findings are provided in Table S6. We examined 2 time points in separate cohorts: immediately following stress exposure (T1) and approximately 1 month later (T2; the time point at which the RNA-seq experiment was performed). As can be seen in Fig. 5d, the expression of miR203-5p increased by approximately 13-fold in mPFC at T1 in C-*Glud1*^+/−^/Stress mice and remained mildly increased at T2 relative to both C-Cre+/Stress and C-Cre+/Control (dotted line) groups. The expression of miR493-5p differed significantly between groups at T1, with higher expression in C-Cre+/Stress mice and marginally higher expression in the C-*Glud1*^+/−^/Stress group; these differences did not persist at T2. Similarly, RT-PCR analysis (n:4-8/group) revealed that several of the DEG transcripts that emerged at T2 were unaltered in the mPFC at T1 (all *p*’s > .239; Table S6).

Differences in the expression of miR137-5p, miR203-3p and miR34a-5p emerged at T2. miR137-5p expression was upregulated in the C-*Glud1*^+/−^/Stress group compared to C-Cre+/Control mice, and marginally compared to the C-Cre+/Stress mice. miR203-3p and miR34a-5p were downregulated in C-*Glud1*^+/−^/Stress mice compared to C-Cre+/Control and C-Cre+/Stress mice. For all 3 miRNA molecules, no differences were found at T1. No differences in miR137-3p or miR493-3p were detected at either time point. The mean miRNA expression score (mean ∆∆Ct across miRNAs for each mouse) at T1 was higher in C-*Glud1*^+/−^/Stress compared to the C-Cre+/Stress (fold change: 2.60 ± 0.24) and C-Cre+/Stress (fold change: 1.21 ± 0.49) mice. No such difference was found at T2.

Analysis of individual miRNA molecules and the mean miRNA expression score at T2 in the dCA1 and vCA1 hippocampal subregions revealed no changes in the C-*Glud1*^+/−^/Stress group. In dCA1, the mean miRNA expression score and the expression of miR137-5p were upregulated in the C-Cre+/Stress group (Fig. S6).

Since mPFC miR203-5p expression increased dramatically at T1, we asked which pathways are associated with targeted transcripts of this miRNA molecule. Pathway analysis revealed targets associated with chromatin regulation, cortisol synthesis, steroid metabolism and glutamate metabolic processes (**5e**).

Finally, we examined the correlations between miRNA expression and behavioral measures in the water T-maze (**5f**; see additional behavioral measures in Table S4). Mice from all 4 groups were included. MiR203-5p expression was correlated with the number of trials to criterion in both phases, but not with variability measures. In contrast, miR34a-5p was correlated with variability measures in both phases, and with trials to criterion in reversal. MiR493-5p was not correlated with any of the measures. Overall, T-maze reversal performance was most highly correlated with GABA, glutamate and stress-related gene expression, as well as with miRNA expression levels, compared to other behavioral measures (Table S4).

## Discussion

Our findings indicate that stress alters the behavioral and molecular phenotype of mice with a constitutive, CNS-specific monoallelic deletion of *Glud1*. Specifically, C-*Glud1*^+/−^ mice exposed to mild stress, which was nearly inconsequential in C-Cre+ controls, exhibit deficits in spatial acquisition and reversal learning, alterations in prefrontal changes in glutamatergic and GABAergic gene expression, and long-lasting changes in miRNA expression. Stress-naive C-*Glud1*^+/−^ mice showed only mild behavioral abnormalities in the open field and relatively limited transcriptomic alterations. Furthermore, we found that spatial discrimination and reversal learning scores correlate with mRNA and miRNA transcription levels in mPFC. These findings are particularly striking considering the mildness of the stressor and the temporal separation (i.e., 3-4 weeks) between stress exposure and behavioral/molecular testing.

Stress-exposed C-*Glud1*^+/−^ mice show deficits in spatial acquisition and reversal in a water T-maze task, unlike their stress-naïve C-*Glud1*^+/−^ and stress-exposed C-Cre+ littermates. We found similar deficits in stress-naïve homozygous C-*Glud1*^−/−^ mice [20], and in WT mice exposed to prolonged social isolation stress in adolescence [34]. Thus, CNS *Glud1* heterozygosity and mild stress have additive effects on cognitive performance in this task. Different manipulations affecting glutamate homeostasis were reported to induce compromised performance in hippocampus-dependent spatial acquisition tasks [51, 52] and mPFC-dependent reversal assays [53–55]. Exposure to acute stress also induces cognitive impairments [56], but its effects in mice with compromised glutamate neurotransmission have not been thoroughly studied. Notably, in our study stress preceded behavioral testing by several weeks. Apparently, the impact of early adulthood stress in our study ‘incubates’ over this time period and leads to additive effects on both the behavioral and molecular levels, in line with previous studies on stress in early life and adolescence [57].

Our findings point to expression changes in 30 genes attributable to the genetic alteration in *Glud1*, and no changes induced by stress exposure alone. However, the combination of *Glud1* deletion and stress resulted in 3,107 DEGs. Many of these genes contribute to homeostasis at the glutamate and GABA synapses, pointing to a potentially disrupted balance between glutamate and GABA transmission in the CNS-*Glud1*^+/−^ /Stress group. Notably, we found no change in *Glud1* mRNA counts in C-*Glud1*^+/−^ mice. However, many genes that interact with *Glud1* were differentially expressed, e.g., the glutamate-glutamine cycle rate-limiting enzyme *Glutaminase 1* (*Gls1*) [58]; *the mitochondrial glutamate oxaloacetate transaminase* (*Got2*) gene which synthesizes glutamate from aspartate and α-ketoglutarate [59], and *Oxoglutarate dehydrogenase (Ogdh)*, a mitochondrial complex member associated with glutamate degradation [60], which regulates neurotransmitter glutamate levels and is implicated in the stress response [61]. Another gene involved in amino acid metabolism altered in C-*Glud1*^+/−^ mice is the *Slc6a18* gene, downregulated roughly 50% in both *Glud1* groups. This gene is part of the *Slc6a* family, involved in transport of compounds related to regulation of inhibitory neural activity [62], and was found to be sodium and chloride dependent [63]. Interestingly, mice with knockout of this gene had higher urine glutamine and glutamate concentrations [63].

While homozygous C-*Glud1*^−/−^ mice show an increase in excitatory transmission under stress-naïve conditions [14], transcriptional abnormalities implicating pyramidal neuron dysfunction in heterozygous CNS-*Glud1*^+/−^ mice are triggered by stress. In CNS-*Glud1*^+/−^/Stress mice, stress may limit the ability of the mPFC to regulate glutamate homeostasis [64, 65], and hinder the competency of the mPFC to implement compensatory mechanisms critical for intact homeostasis at the glutamate tripartite and glutamate-GABA synapses. In particular, the present findings point to changes in pyramidal cells, GABA interneurons and oligodendrocytes. The function of these cell types and their interactions are disrupted in several psychiatric disorders including schizophrenia. Post-mortem PFC transcriptional data from schizophrenia patients reveals changes in pathways associated with synaptic signaling [66], glutamate signaling [66, 67], the GABAergic synapse and postsynaptic membrane [68]. Oligodendrocytic deficits could point to myelination deficits in excitatory or inhibitory neurons. In turn, dysfunctional excitatory /inhibitory neurons could impact NMDA receptors located on oligodendrocytes, thus affecting their function [69]. The association between excitatory/inhibitory neurons and oligodendrocytes was shown in human studies [70, 71] and in animal models of cognitive dysfunction [72].

Epigenetic processes may account for the unique impact of stress on the transcriptional profile of C-*Glud1*^+/−^/Stress mice. Stress was shown to impair cognitive function and alter the expression of glutamate- and GABA-related genes via epigenetic changes to DNA or RNA [73–76]. Here, we show that the expression of the stress-associated miRNA molecule miR203-5p, which targets glutamate, stress and epigenetic-associated transcriptional pathways, is upregulated immediately after stress, and remains elevated several weeks later. One possibility is that changes in miR203-5p (and/or other miRNA molecules not examined in the present study) lead to transcriptional changes, potentially through other epigenetic mechanisms such as histone methylation/acetylation and heterochromatin modifications, that escalate over time and result in impaired glutamate homeostasis and glutamate-GABA communication.

Interestingly, stress led to miRNA expression changes in dCA1 in control (Cre+), but not C-*Glud1*^+/−^ mice. Spatial rule learning depends on dorsal hippocampal function, and was impaired in C- *Glud1*^+/−^ mice. Taken together with the mPFC findings, these data point to a dichotomous miRNA-behavior relationship in dCA1 vs. the mPFC: in dCA1, elevated miRNA expression signifies intact spatial learning, whereas in the mPFC it signals cognitive impairment.

Performance level in the T-maze reversal task was correlated with the expression level of glutamate, GABA and several stress-related genes. Previous investigations found that performance in the Morris Water Maze, a hippocampus-dependent spatial acquisition task, is correlated with hippocampal gene expression, particularly affecting the homeostasis of glutamatergic synapses [77]. Here, we further find that genes contributing to glutamate homeostasis, e.g. the mitochondrial glutamate carrier 1 gene, *Slc25a22*, as well as the schizophrenia risk gene *Grin1* [78], distinctly differentiate between high- and low-performers in mPFC-dependent reversal learning [31]. This further supports the importance of glutamate homeostasis, maintained by a multitude of metabolic and synaptic components, in cognitive function.

This is the first demonstration of a gene x environment interaction in mice with abnormal expression of *Glud1*, which is downregulated in schizophrenia [20] and predicts antipsychotic treatment efficacy [79]. Similarly to other psychiatric disorders, schizophrenia symptoms are believed to be ‘triggered’ by an external event in individuals with underlying genetic susceptibility [80]. Cognitive symptoms precede and often predict the eruption of the first psychotic episode [81]. Thus, stress-exposed C-*Glud1*^+/−^ mice may provide a tool for studying high-risk populations which carry genetic susceptibility and may be particularly vulnerable to the effects of adversity.

## Methods

### Mice

C-*Glud1*^−/−^ mice were bred at the University of Haifa mouse vivarium. Flx-*Glud1* (*Glud1*^lox/lox^) mice (kind gift from Pierre Maechler, University of Geneva) were bred with mice expressing *Cre* recombinase under the control of the *Nestin* cis-regulatory sequence (Jackson labs, Sacramento CA) to generate *Nestin-Cre*::*Glud1*^lox/+^ (C-*Glud1*^+/−^) mice. *Nestin-Cre*::*Glud1*^+/+^ (C-Cre+) mice were used as controls, since they were found to display mild physiological and behavioral differences compared to Cre- mice [14]. Mice were maintained on a C57BL/6 J background. Procedures involving mice and their care were conducted in conformity with the National Institutes of Health Guide for the Care and Use of Laboratory Animals, under approval by the University of Haifa Ethics and Animal Health Committee (587/18). Food and water were provided ad libitum. Experiments included approximately equal numbers of males and females per group. The sample size was determined based on a priori power analysis using G*Power software using a moderate effect size at an alpha error probability of 0.05 [82].

### Procedure

On postnatal day (PND) 70, male and female C-Cre+ and C-*Glud1*^+/−^ mice were randomly divided into Stress or Control Groups. The experiment thus consisted of 4 groups: C-Cre+/Control, C-Cre+/Stress, C-*Glud1*^+/−^/Control and C-*Glud1*^+/−^/Stress. Acute mild physical stress consisted of 3 i.p. injections of 0.9% saline within a 24-hr period: at time 0 (14:00), 21 h later and 23 h later [37]. Control (Stress-naïve) mice were held, but no injections were performed. The injection location, lighting conditions, and time were kept constant across mice, and the same experimenter administered injections to all mice. This stress protocol was previously shown to disrupt behavior and increase corticosterone levels a few hours after the stress procedure [37]. Here, we assessed behavioral and molecular phenotypes starting a week after the last injection (PND 77). The behavioral assessment consisted of the following tests: nesting, open field, social preference, social recognition, and spatial discrimination (acquisition) and reversal learning in the water T-maze [14, 34]. Mice from each group were randomly selected for mRNA/miRNA expression analysis, and were sacrificed by cervical dislocation on PND 98. A separate cohort of mice underwent the same stress procedure followed by brain removal immediately after the last i.p. injection, for examination of miRNA expression immediately following stress.

### Behavioral tests

Behavioral assays are described in detail in the SI.

Briefly, *Nesting* assesses the ability of male and female rodents to construct a nest in their home environment. Disrupted nesting behavior is a correlate of anhedonia and self-neglect [83]. Nest quality was evaluated by 2 independent, condition-blind raters on a 1–5 scale [84]. The nesting score was calculated as the mean of the raters’ scores.

The *Open field* test examines spontaneous locomotion, anxiety-like behavior, and the ability to adapt to a novel location [85, 86]. Mice were put in a white unfamiliar plexiglass arena for 60 min. Trials were recorded and analyzed using Ethovision XT14.0 software (Noldus Information Technology, Leeburh, VA). Outcome measures were the distance traveled (cm; total and in each 5-min bin) and the time (sec; total and in each bin) spent in the center of the arena.

The *Social preference and recognition* tests assess social behavior and memory [87]. Briefly, mice were tested in a 3-chamber arena equipped with clear plexiglass cages. Mice were first tested for their preference of an unfamiliar mouse vs. a novel object. After a 60 min ITI, mice were returned to the arena and tested for recognition of novel vs. familiar social stimuli. Outcome measures were the exploration time of each stimulus, and the preference ratio (time exploring the social/total exploration time) and recognition ratio (time exploring the novel mouse/total exploration time) during the preference and recognition phases, respectively [14, 34]. Trials were recorded and analyzed using Ethovision.

Spatial rule acquisition and reversal learning were assessed in the modified *Water T-maze* task [88]. Mice were placed in a gray plexiglass T-shaped maze filled with water (25 °C ± 1). Following an arm preference test (3 trials), mice were trained to swim to an underwater platform located in the less-preferred arm, until a criterion of 5 consecutive correct choices was achieved (Acquisition phase). After a 3-min ITI, a probe test was conducted, and the platform was placed in the opposite arm. Mice were tested until a 5 consecutive correct trial criterion was reached (Reversal). Outcome measures for each phase were: (i) the number of trials to criterion, (ii) the reaction time (RT: time (sec) to reach the end of any arm), and (iii) RT variability (RTv: the standard deviation of the differences in RTs between each pair of successive trials, per mouse. A higher number of trials to criterion, longer RTs and higher RTv reflect compromised performance.

### mRNA and miRNA expression analysis

Mice were sacrificed 14 days after the end of the behavioral battery (PND 98) by cervical dislocation. The mPFC, consisting of the prelimbic and infralimbic subregions, and the dorsal and ventral CA1 subregions of the hippocampus (dCA1 and vCA1, respectively), were removed bilaterally using 0.5 mm punches [89].

### Genome wide RNA-Seq

Full procedural details are provided in the SI. RNA was extracted from 28 mPFC samples (*n* = 5–9 per group, approximately equal numbers of males and females) as previously described [20, 90], and sent to the Technion Genome Center for genome-wide RNA sequencing (RNA-seq) and bioinformatical analysis. The experiment was comprised of two batches; the first included samples from three of the four groups (C-Cre+/Control, C-Cre+/Stress and C-*Glud1*^+/−^/Stress), and the second included new samples from the three aforementioned groups, and added the forth group (C-*Glud1*^+/−^/Control). Findings from both batches were combined, taking into account batch and sex effects. RNA was prepared using the SMARTer Stranded Total RNA-Seq Kit v2 – Pico preparation kit according to the manufacturer’s instructions. RNA-seq library preparation, sequencing (using the Illumina HiSeq 2500 sequencer for first batch; Illumina NextSeq 550 for the second), and data analysis was performed by the Technion Genome Center. The quality of the libraries was evaluated using FASTQC (v 0.11.5), quality and adapter trimming was conducted via trim galore (uses cutadapt v 1.10), and mapping was conducted via Tophat2 v 2.1.0, (uses short read aligner Bowtie2 v 2.2.6). At the end of this process, the total reads after trimming ranged between 35–46 million reads per sample. Gene counting was conducted via HTseq-count (v0.6.1). Due to the ribosomal depletion process in the library preparation protocol and the expected ribosomal sequence reads, counting was performed with a modified annotation file which includes the 45 s ribosome annotation for better accuracy. Only counted reads without 45 s were used for the subsequent analysis. Uniquely mapped reads, aligned with high confidence to a single genomic location, ranged between 25–38 million reads per sample. Differential gene expression was performed by DESeq2 (v1.28.1).

### MiRNA /mRNA expression analysis

The expression of MiR137-3p, MiR137-5p, MiR34a-5p, MiR203-3p, MiR203-5p, MiR493-3p and MiR493-5p and mRNA of 4 select DEGs were assessed by quantitative reverse transcription PCR (qRT-PCR). Reactions were carried out using a StepOne qRT-PCR system (Applied Biosystems). Fold-change values were calculated using the ∆∆Ct method [91] relative to the housekeeping genes RNU6 and RNU66 (miRNA) or HPRT (mRNA). A mean expression score for all 7 miRNAs was calculated as the mean ∆∆Ct value across miRNAs for each mouse.

### Statistical analysis

Full description of statistical tests is provided in the SI. Statistical analyses were performed using SPSS 27 software (IBM SPSS Statistics, New York, United States), Prism 8 (GraphPad software, Inc.) or jamovi v 2.2.5, following normality and homogeneity of variances assumptions verification. Factorial analysis (ANOVA) was applied to behavioral data. Male and female data were analyzed separately in cases of a Sex main effect or interaction with the Sex variable. Significant interactions were followed by LSD post hoc comparisons.

In the RNA-seq experiment, normalization and differential expression analysis of uniquely mapped reads were conducted using the DESeq2 R package (v1.28.1). Three paired comparisons were conducted (C-Cre+/Stress, C-*Glud1*^+/−^/Control and C-*Glud1*^+/−^/Stress were each compared to C-Cre+/Controls) using negative binomial GLM. An FDR correction [92] was computed (*q* < 0.05): only genes with an FDR-corrected *q* value lower than 0.05 were considered statistically significant. Over-representation analysis (ORA), analysis of functional networks of protein-protein interactions, transcript cluster analysis, and correlations were performed (see SI and Results for details).

miRNA and mRNA data were analyzed using non-parametric Kruskal–Wallis tests, with Monte Carlo simulation for exact *p* values. Dunn tests were used to test post-hoc comparisons. Correlations between transcriptional and behavioral data were FDR-corrected (*q* < 0.05).

## Supplementary information

Supplementary Material Methods and Results

Table S2

Table S3

## Data Availability

The RNA-seq data from this study are available at 10.5061/dryad.6q573n63k.
