# Supplementary material for: Synergistic, long-term effects of glutamate dehydrogenase 1 deficiency and mild stress on cognitive function and mPFC gene and miRNA expression
Source: Transl Psychiatry. 2023 Jul 7;13:248. doi: 10.1038/s41398-023-02534-y (PMC10328942; doi:10.1038/s41398-023-02534-y)
Supplement: Supplementary file 1 — Supplementary Material Methods and Results [file 41398_2023_2534_MOESM1_ESM.docx]

Supplementary Information (SI)

**Method**

*Behavioral Tests*

The behavioral battery consisted of the following tests: nesting, open field, social preference, social recognition, and the water T-maze task which included spatial discrimination (acquisition) and reversal phases^1,2^. The order of testing was determined such that naturalistic, relatively non-aversive tests (i.e., nesting, open field, social preference and recognition) preceded the aversive water T-maze task, based on previous studies in our lab^1,2^. The *Nesting* assay assesses the ability of male and female rodents to construct a nest given the appropriate materials in their home environment. Disrupted nesting behavior is a correlate of anhedonia and self-neglect, and is indicative of disrupted hippocampal function^3^. Nest quality was evaluated by 2 independent, condition-blind raters on a 1-5 scale^4^. The nesting score was calculated as the mean of the raters’ scores [ICC=.889, CI: .839-.923 (Absolute agreement)].

The *Open field* test examines spontaneous locomotion, anxiety-like behavior, and the ability to adapt to a novel location^5,6^. Mice were placed in a white unfamiliar plexiglass arena (50 L*50 W*40 H, cm) for 60 min. Trials were recorded and analyzed using Ethovision XT14.0 software (Noldus Information Technology, Leeburh, VA). Data were divided into 5-min bins. Outcome measures were the distance travelled (cm; total and in each bin) and the time spent in the center of the arena (sec; total and in each bin; 30 W * 25H, cm).

The *Social preference and recognition* tests examine social behavior and memory^7^. Mice were placed in the center chamber of a three-chamber arena (60L * 40W * 25H, cm) equipped with two square, transparent Plexiglass cages in each side chamber. Mice were allowed to freely explore the entire arena for a 10 minutes period. For the Social Preference test, an unfamiliar, same-sexed, and younger mouse was placed in one of the cages (the “social chamber”), while the other (“neutral”) chamber remained vacant. After a 60 min ITI in a holding cage, mice were returned to the arena for a 10 minutes Social Recognition test. For this test, a familiar and novel mouse were each placed in the cages, with their location relative to the previous stage counterbalanced across mice. Outcome measures were the preference ratio (time exploring the social chamber/total exploration time) and recognition ratio (time exploring the novel mouse/total exploration time) during the preference and recognition phases, respectively^1,8^. Trials were recorded and analyzed using Ethovision software.

*Water T-maze* – Spatial rule acquisition and reversal^9^ were assessed using the Water T-maze as previously described^1^. A grey plexiglass T-shaped maze (stem 64L * 13W * 43H, arms 42L * 13W * 43H, cm) was mounted 40 cm above the floor with minimal extra-maze visual cues. Sliding doors, which were manually opened or closed, were located 1 cm from the opening of each arm. The maze was filled with water (25°C ± 1), approximately 26 cm deep. A removable transparent plexiglass platform (12 * 12, 25H, cm) was located 1 cm below the water level at the end of one of the arms. The task consisted of 2 phases.

In the first phase (Acquisition), mice were tested for arm preference (3 trials) and trained to swim to a platform located in the less-preferred arm, until a criterion of 5 consecutive correct choices was achieved. If the correct arm was chosen, the mouse was allowed to rest on the platform for 10 s. If the incorrect arm was chosen, the mouse was immediately removed from the maze. If a mouse chose the wrong arm 10 times in a row, a sliding door was closed, and the mouse was confined to that arm for 20s. Following each trial, the mouse was removed from the maze to a holding cage for a 10 s ITI. Mice were trained to a criterion of 5 consecutive correct trials, with a maximum of 30 trials per day. Mice that successfully reached criterion were given a rest period for 3 minutes. Then, a probe test was conducted, with no platform in the maze; mice that did not choose the previously-rewarded arm were re-trained on the next day. Mice that passed the probe test were given a 10s ITI and proceeded to the next phase of the experiment. In the second phase (Reversal), the platform was moved to the opposite arm, and mice were trained to a criterion of 5 consecutive correct trials, with a maximum of 30 trials per day.

Outcome measures for each phase were: (i) the number of trials to criterion, (ii) the reaction time (RT: time (sec) from beginning of the trial until the mouse reaches the end of any arm), and (iii) RT variability (RTv), i.e., the standard deviation of the differences in RTs between each pair of successive trials, per mouse. While the number of trials to criterion is a commonly used outcome measure^10–12^, RT and RT variability contribute to a broader understanding of the mice performance. RT provides a measure of motivation^13,14^, whereas RTv provides a measure of performance consistency and stability^15,16^ Since performance normally improves during the learning progress, becoming more stable and consistent, longer RTs and higher RTv reflect compromised learning and more erratic behavior.

*RNA-seq*

RNA was extracted as previously described^17,18^. Briefly, dissected brain regions were homogenized in 300 µL of TRIzol Reagent (Invitrogen, Carlsbad, CA) and 10 μl glycogen (Sigma-Aldrich), then suspended in a total of 0.5 ml TRIzol. After adding 200 μl chloroform to allow phase separation by centrifugation (14,000 rpm, 15', 4 °C), 250 μl isopropanol (Sigma-Aldrich) was added to the aqueous phase. After a night in −20^o^C, the RNA was precipitated by centrifugation (14,000 rpm, 15', 4 °C). The pellet was washed in 500 μl cold 100% ethanol and centrifuged (7,600 rpm, 10'), and then dried. RNA was diluted in 20 µl water and quantities were determined using a Nanodrop 2000 spectrophotometer (Thermo Scientific, Wilmington, DE, USA). The 260:280 nm absorbance ratio was measured to assess RNA quality; samples were excluded if the ratio was outside the range of 1.7-2.0, or if RNA concentration was too low. DNA digestion was carried out with 1 μL DNase (Fermentas), incubated at 37 °C for 30 minutes, and then deactivated at 65 °C for 10 minutes. RNA measurements, quality assessment, library preparation, sequencing and data normalization were performed by the Technion Genome Center. RNA concentration was measured using a Qubit 4 Fluorometer (ThermoFisher Scientific). RNA quality was measured using Agilent 2200 TapeStation (Agilent, Santa Clara, CA, USA). Libraries were prepared using the SMARTer Stranded Total RNA-Seq Kit v2 - Pico Input Mammalian (Takara). These libraries were sequenced using the Illumina HiSeq 2500 sequencer (batch 1) or Illumina NextSeq 550 (batch 2). The quality of the libraries was evaluated using FASTQC (v 0.11.5), quality and adapter trimming was conducted via trim galore (uses cutadapt v 1.10), and mapping was conducted via Tophat2 v 2.1.0, (uses short read aligner Bowtie2 v 2.2.6). At the end of this process, the total reads after trimming ranged between 35-46 million reads per sample. Gene counting was conducted via HTseq-count (v0.6.1). Due to the ribosomal depletion process in the library preparation protocol and the expected ribosomal sequence reads, counting was performed with a modified annotation file which includes the 45s ribosome annotation for better accuracy. Only counted reads lacking 45s were used for the subsequent analysis. Uniquely mapped reads, aligned with high confidence to a single genomic location, ranged between 25-38 million base pairs per sample. Differential gene expression was performed by DESeq2 (v1.28.1).

*miRNA and mRNA expression analysis*

RNA extraction was performed as described above and quantities were determined using a Nanodrop 2000 spectrophotometer (Thermo Scientific). For mRNA, DNA digestion was carried out with 1 μL DNase (Fermentas), incubated at 37 °C for 30 minutes, and then deactivated at 65 °C for 10 minutes. Reverse transcription was carried out using qScript cDNA Synthesis Kit (Quanta Biosciences, Gaithersburg, USA) following the manufacturer’s protocol. cDNA was amplified in a 10 µL reaction (0.6 µL primers (10µM), 5 µL PerfeCTA SYBR green (Quanta Biosciences, Gaithersburg, USA), 2 µL cDNA (diluted 1:9 with H2O) and 2.4 µL ddH2O), by a Step One RT-PCR machine (Applied Biosystems, Carlsbad, CA). Primers for *Gls1* (F: 5'-GCA-CTA-CAC-TTT-GGA-CAC-CA-3'; R: 5'-ATG-TTC-CAC-AAT-GAC-TAG-GTA-AGT-3'), *Grin1* (F: 5'-CAG-GAG-CGG-GTA-AAC-AAC-AGC-AAC-3'; R: 5'-GAC-AGC-CCC-ACC-AGC-AGC-CAC-AGT-3')*, Nr3c1* (F: 5'-TTC-TAA-TGG-ATA-TTC-AAG-CCT-GG-3'; R: 5'-TGG-ACG-GAG-GAG-AAC-TCA-CAT-3') and *Vglut1* (F: 5'-GGT-GCA-GAA-AGC-CCA-GTT-CA-3'; R: 5'-AGT-GAC-AAT-GTA-GCC-CCA-GAA-AAA-3') were either designed using *Primer3* software^19^ and synthesized by Integrated DNA Technologies (Coralville, IA), or designed and synthesized by Agentek (Tel Aviv, Israel). Primer suitability was determined using standard curve analysis, melting curve analysis, and linearity at threshold^20^.

miRNA expression analysis was carried out as described^18^. Briefly, reverse transcription reactions were performed using the qScript microRNA cDNA Synthesis Kit (Quanta Biosciences, Gaithersburg, USA) following the manufacturer’s protocol. The expression of MiR137-3p, MiR137-5p, MiR34a-5p, MiR203-3p, MiR203-5p, MiR493-3p and MiR493-5p were assessed using SYBR Green qRT-PCR amplification (5 ng total RNA in 20 μl total reaction volume) using specific primers (0.4 μl each, Quanta Biosciences, Gaithersburg, USA) according to manufacturer’s instructions. qRT-PCR reactions were carried out using a Step One real-time PCR system (Applied Biosystems). Fold-change values were calculated using the ∆∆Ct method^21^ relative to the housekeeping genes RNU6 and RNU66 (miRNA) or HPRT (mRNA; F: 5'-CGC-CAG-CTT-CCT-CCT-CAG-3'; R: 5'-ATA-ACC-TGG-TTC-ATC-ATC-ACT-AAT-CAC-3').

*Statistical analysis*

Behavioral data were analyzed following verification of the normality assumption using the Kolmogorov-Smirnov and Shapiro-Wilk tests, and after skewness and kurtosis values were examined (value lower than |2| was considered acceptable). The variances were approximately homogeneous between the groups that were statistically compared. This was verified by (visual inspection and) relevant statistical assays, e.g., Levene's and Bartlett's tests. Factorial analysis (a one-way ANOVA, factorial between-subjects ANOVA or a factorial repeated-measures ANOVA, as appropriate) were performed. In case of a significant effect involving Sex, data were analyzed separately for males and females. Otherwise, data from males and females were pooled. Sphericity correction was applied when appropriate. Significant interactions were followed by t-test post hoc comparisons. The effect size estimator for ANOVA effects was partial eta-squared (η²_p_). STRING^22^ was used to examine protein-protein interactions and gene clusters (Interaction score > .400, considered medium confidence; Inflation parameter = 3). We used the DIANA miRNA database to search for targets of annotated miRNAs. This tool uses the popular Micro-CDS algorithm^23^ (Genes with target prediction score above 0.60) and compares it to the TargetScan database^24^.

**Results**

| Assay | Measure/ Fig # | Statistical test | Effects/Interaction/post-hocs |  |
| --- | --- | --- | --- | --- |
| Nesting | Nest Building Quality | 2-way ANOVA | Genotype: F_(1,68)_=0.68, *p*=.411, η²_p_=.009  Group: F_(1,68)_=0.43, *p*=.512, η²_p_=.006  Genotype * Group: F_(1,68)_=0.29, *p*=.591, η²_p_=.004 |  |
|  |  |  |  |  |
|  |  |  |  |  |
| Open field | **Males and Females:** Total distance traveled | 4-way repeated measures ANOVA | **Sex: F_(1,105)_=8.60, *p*=.004, η²_p_=.075** |  |
|  | **Males and Females:** Time in center | 4-way repeated measures ANOVA | **Time * Sex: F_(6.10,640.82)_=2.35, *p*=.028, η²_p_=.021** |  |
|  | **Males:** Total distance traveled | 3-way repeated measures ANOVA | **Time: F_(4.67,233.60)_=71.20, *p*<.001, η²_p_=.587**  **Genotype: F_(1,50)_=5.57, *p*=.022, η²_p_=.100**  Group: F_(1,50)_=0.13, *p*=.711, η²_p_=.002  Genotype * Group: F_(1,50)_=0.11, *p*=.739, η²_p_=.002  **Time * Genotype: F_(4.67,233.60)_=3.46, *p*=.005, η²_p_=.064 Post hoc tests: Bin 1 *p*=.052; bins 2-8 *p*’s<.05** |  |
|  |  |  |  |  |
|  |  |  |  |  |
|  |  |  |  |  |
|  |  |  |  |  |
|  |  |  |  |  |
|  | **Females:** Total distance traveled | Three-way repeated measures ANOVA | **Time: F_(5.83,320.81)_=91.41, *p*<.001, η²_p_=.624**  Genotype: F_(1,55)_=0.91, *p*=.341, η²_p_=.016  Group: F_(1,55)_=1.34, *p*=.251, η²_p_=.023  Time * Genotype: F_(5.83,320.81)_=1.14, *p*=.336, η²_p_=.020  Genotype * Group: F_(1,55)_=1.66, *p*=.202, η²_p_=.029 |  |
|  |  |  |  |  |
|  |  |  |  |  |
|  |  |  |  |  |
|  |  |  |  |  |
|  |  |  |  |  |
|  | **Males:** Time in center  Time in center, Bin 1 | Three-way repeated measures ANOVA  Two-way ANOVA | **Time: F_(6.16,308.46)_=2.87, *p*=.009, η²_p_=.054**  **Time * Genotype: F_(6.16,308.46)_=2.68, *p*=.013, η²_p_=.051** C-Cre+: F_(5.98,191.63)_=2.09, *p*=.056  C-Glud1+/-: F_(5.25,105.06)_=3.69, *p*=.003, η²p=.155  **Linear trend: F_(1, 20)_=8.08, *p*=.010, η²p=.287**  **Polynomial trend: F_(1, 20)_=6.37, *p*=.020, η²p=.241(order 5), F_(1, 20)_=5.09, *p*=.035, η²p=.203 (order 9)**  **Genotype: F_(1,50)_=4.49, *p*=.038, η²_p_=.082**  **Group: F_(1,50)_=8.47, *p*=.005, η²_p_=.144**  Genotype * Group: F_(1,50)_=0.12, *p*=.723, η²_p_=.002 |  |
|  |  |  |  |  |
|  |  |  |  |  |
|  |  |  |  |  |
|  |  |  |  |  |
|  |  |  |  |  |
|  |  |  |  |  |
|  |  |  |  |  |
|  |  |  |  |  |
|  |  |  |  |  |
|  |  |  |  |  |
|  |  |  |  |  |
|  |  |  |  |  |
|  |  |  |  |  |
|  |  |  |  |  |
|  |  |  |  |  |
|  |  |  |  |  |
|  |  |  |  |  |
|  | **Females:** Time in center  Time in center, Bin 1 | Three-way repeated measures ANOVA  Two-way ANOVA | **Time: F_(5.26,289.34)_=3.33, *p*=.005, η²_p_=.057**  Time * Genotype: F_(5.26,289.34)_=1.05, *p*=.389, η²_p_=.018  **Genotype: F_(1,55)_=6.64, *p*=.012, η²p=.107**  **Group: F_(1,55)_=8.96, *p*=.004, η²p=.140**  Genotype * Group: F_(1,55)_=0.70, *p*=.404, η²p=.012 |  |
|  |  |  |  |  |
|  |  |  |  |  |
|  |  |  |  |  |
| Social preference | Social preference ratio | One-sample t-test (μ=0.5) | ***p*'s<.05 for all 4 groups. All means > 0.5** |  |
|  |  | Two-way ANOVA | Genotype: F_(1,80)_=3.11, *p*=.081, η²_p_=.037  Group: F_(1,80)_=1.23, *p*=.269, η²_p_=.015  Genotype * Group: F_(1,80)_=1.70, *p*=.195, η²_p_=.020 |  |
|  |  |  |  |  |
|  |  |  |  |  |
| Social recognition | Social recognition ratio | One-sample t-test (μ=0.5) | *p*'s>.05 for all 4 groups. All means <=0.5 |  |
|  |  | Two-way ANOVA | Genotype: F_(1,80)_=2.79, *p*=.098, η²_p_=.033  Group: F_(1,80)_=0.42, *p*=.516, η²_p_=.005  Genotype * Group: F_(1,80)_=1.19, *p*=.278, η²_p_=.014 |  |
|  |  |  |  |  |
|  |  |  |  |  |
| Water T-maze | Acquisition:Trials to criterion | Two-way ANOVA | **Genotype * Group: F_(1,90)_=12.68, *p*<.001, η²_p_=.122**  C-*Glud1*^+/-^/ Stress > all other groups (*p*’s<.001) |  |
|  | Acquisition: Reaction Time (RT) | Two-way ANOVA | **Genotype * Group: F_(1,90)_=7.97, *p*=.006, η²_p_=.081**  C-*Glud1*^+/-^/ Stress> all other groups (*p*’s<.001) |  |
|  | Acquisitio: RT variability (RTv) | Two-way ANOVA | **Genotype * Group: F_(1,90)_=4.94, *p*=.029, η²_p_=.052**  C-*Glud1*^+/-^/ Stress>all other groups (*p*’s<.005) |  |
|  | Acquisition phase - Failure to achieve criterion | Fisher's exact test (Monte-Carlo simulated) | Proportion failing to achieve criterion: **C-*Glud1*^+/-^/ Stress>all other groups (*p*=.001)** |  |
|  | Reversal: Trials to criterion | Two-way ANOVA | **Genotype * Group: F_(1,87)_=17.79, *p*<.001, η²_p_=.170**  C-*Glud1*^+/-^/ Stress> all other groups (*p*’s<.001) |  |
|  | Reversal: RT | Two-way ANOVA | **Genotype * Group: F_(1,87)_=15.31, *p*<.001, η²_p_=.150**  C-*Glud1*^+/-^/ Stress> all other groups (*p*’s<.001) |  |
|  | Reversal: RTv | Two-way ANOVA | **Genotype * Group: F_(1,87)_=34.05, *p*<.001, η²_p_=.281**  C-*Glud1*^+/-^/ Stress> all other groups (*p*’s<.001) |  |

**Table S1**: **Behavioral tests: statistical analyses**

*Nesting –* Sex groups were pooled, as no Sex main effect or interactions were found. No main effects for Genotype, Group, or an interaction between the groups were found (**Fig S2a**).

*Social preference and social recognition –* Sex groups were pooled, as no Sex main effect or interactions were found. One male from the stress-naïve C-Cre+ group was excluded from analyses (statistical outlier: >2SD above group mean).

*Social preference* – Sex groups were pooled, as no Sex main effect or interactions were found. The social preference ratio was greater than 0.5 in all 4 groups, indicating that all mice spent more time near the social vs. inanimate stimulus (**S1b**). No main effects of Genotype, Group, or an interaction between the groups were found.

*Social recognition* – The social recognition ratio was not different from 0.5 in all 4 groups, pointing to inadequate recognition of the novel vs. familiar social stimulus (**S1c**). No main effects for Genotype, Group, or an interaction between them were found.

**Figure S1: Behavior in the Nesting and Social Preference/Recognition paradigms.** C-Cre+ and C-*Glud1*+/- mice exhibit similar nesting (**a**) and social preference (**a**) regardless of stress exposure; no social recognition was detected in any of the groups (**c**). **^** ratio significantly greater than 0.5; one-sample t-test, all *p*'s<.05.

**Figure S2:** **Higher Individual RT Variability at reversal in C-*Glud1*^+/-^/ Stress mice**. Representative data of mice (n=1) from C-Cre+/ Control and C-*Glud1*^+/-^/Stress groups. Inter-trial RT variability for the C-Cre+/ Control mouse and C-*Glud1*^+/-^/Stress mice across trials: SD=4.83 and SD=45.06, respectively.

**Figure S3: Over-representation analysis of DEGs in mPFC of C-*Glud1* ^+/-^/Stress mice.** Glutamatergic, GABAergic and stress-related pathways are over-represented in C-*Glud1* ^+/-^ /Stress mice according to GO Biological Process (**a**) and Elsevier Pathway Collection (**b**). Combined score is calculated as log(*p* value)*Z score.

**Figure S4:** **Abnormal expression of glutamatergic, GABAergic and Stress-related genes in mPFC of Stress-Exposed C-*Glud1*^+/-^ Mice. (a-c)** shows comparisons between C-Cre+ Stress (left; n=7), C-*Glud1*^+/-^ Control (middle; n=5) and C-*Glud1*^+/-^ Stress mice (right; n=9) in the mPFC expression of genes related to glutamate (**a**) and GABA (**b**). C-Cre+ Control mice (n=7) are the reference group. All genes appear in **a-b** are statistically significant exclusively in the C-*Glud1*^+/-^ /Stress group (*q*<.05).


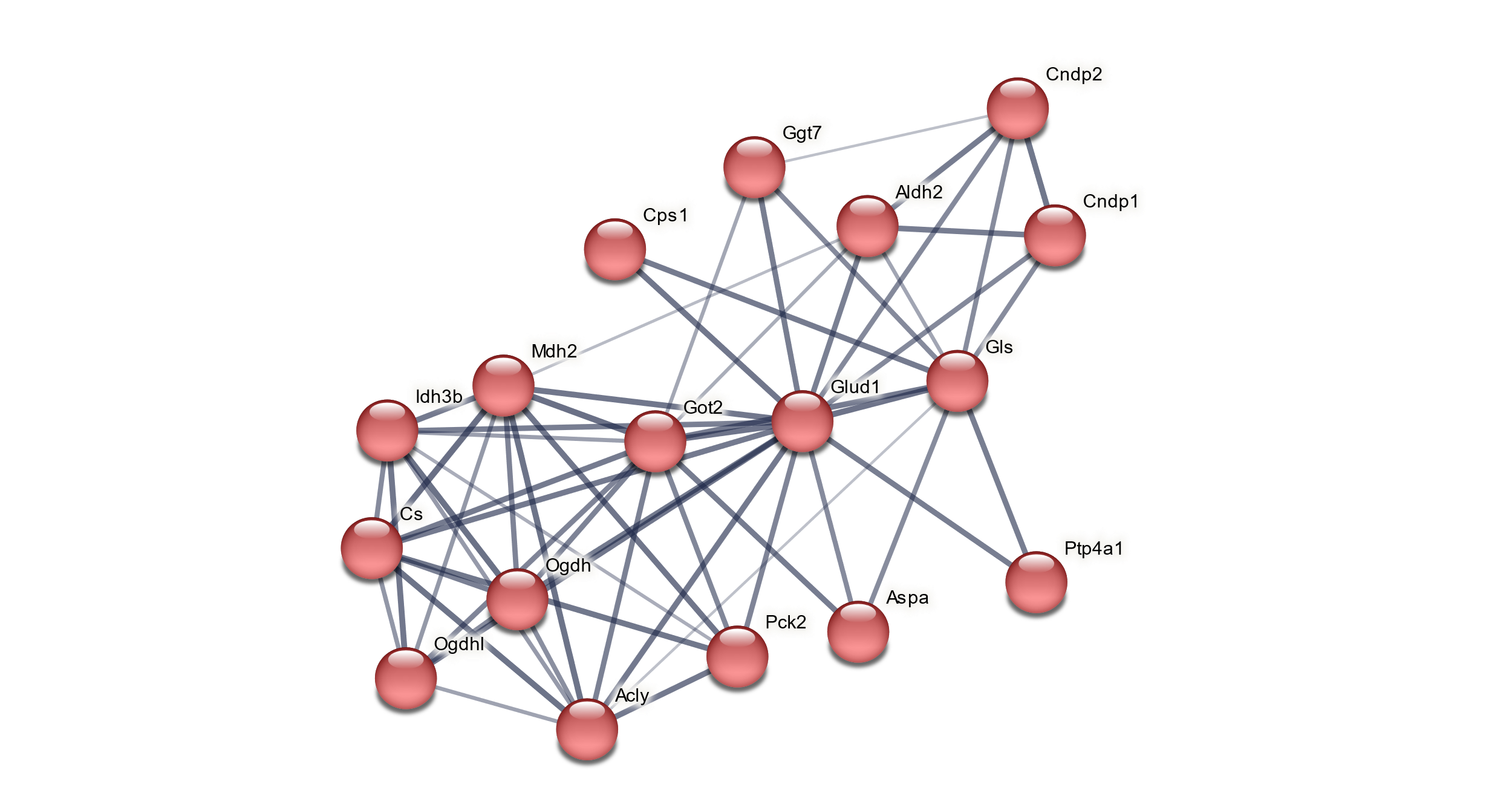


**Figure S5:** **Protein-protein interactions associated with *Glud1***. Functional diagram of protein- protein interaction of *Glud1*-related DEG-encoded proteins in C-*Glud1* ^+/-^ stressed mice, compared to C-Cre+ Control mice. Thicker line indicates stronger support for the interaction.

| Gene | OF: Distance travelled Bin 1 | OF: Total Distance travelled | OF: Time in center Bin 1 | OF: Total time in center | WTM: Trials to criterion Acquisition | WTM: Trials to criterion Reversal | WTM: RTv Acquisition | WTM: RTv Reversal |
| --- | --- | --- | --- | --- | --- | --- | --- | --- |
| *Slc32a1* | -.336 | .219 | -.334 | -.309 | .227 | .501** | .289 | .700** |
| *Gabbr1* | -.068 | .178 | -.422 | -.418 | .352 | .436* | .485* | .701** |
| *Gabrb1* | .108 | .010 | .456* | .492* | -.440* | -.438* | -.561** | -.657** |
| *Gabrd* | -.338 | .040 | -.130 | -.168 | .387* | .308 | .392* | .544** |
| *Gabrg1* | .092 | -.131 | .294 | .475* | -.332 | -.580** | -.308 | -.654** |
| *Nr3c1* | .105 | -.034 | .536* | .514* | -.332 | -.437* | -.450* | -.600** |
| *Nr3c2* | .001 | -.003 | .603** | .286 | -.308 | -.274 | -.708** | -.428* |
| *Slc12a2* | -.022 | .139 | .496* | .439* | -.551** | -.405* | -.531** | -.560** |
| *Slc12a5* | -.205 | .078 | -.338 | -.264 | .362 | .286 | .644** | .447* |
| *Aspa* | -.151 | .222 | .434* | .095 | -.291 | -.063 | -.342 | -.183 |
| *Camk2n2* | -.284 | -.073 | -.451* | -.327 | .367 | .445* | .540** | .683** |
| *Doc2a* | -.049 | .055 | -.514* | -.496* | .389* | .327 | .558** | .729** |
| *Gls1* | .265 | -.017 | .309 | .423 | -.269 | -.530** | -.323 | -.475* |
| *Got2* | -.027 | .382 | -.270 | -.118 | -.020 | .275 | .224 | .561** |
| *Grik1* | -.099 | -.255 | .490* | .414 | -.441* | -.321 | -.458* | -.467* |
| *Grik2* | .134 | -.100 | .399 | .482* | -.297 | -.398* | -.521** | -.581** |
| *Grik5* | -.232 | -.012 | -.503* | -.634** | .309 | .569** | .125 | .690** |
| *Grin1* | -.166 | .123 | -.464* | -.531* | .367 | .392* | .447* | .777** |
| *Slc25a22* | -.157 | .143 | -.471* | -.443* | .306 | .359 | .429* | .701** |
| *Slc38a7* | -.222 | .175 | -.569** | -.438* | .149 | .326 | .241 | .460* |
| *Slc17a7* | -.179 | .161 | -.468* | -.503* | .331 | .424* | .464* | .760** |
| *slc25a13* | .121 | .162 | .439* | .068 | -.322 | -.136 | -.643** | -.201 |
| MiR137-3p | -.117 | -.367 | .083 | .300 | .305 | .033 | .258 | .093 |
| MiR137-5p | .624 | -.552 | -.442 | .212 | .307 | .769** | .698** | .753** |
| MiR34a-5p | -.683* | .283 | .467 | -.017 | -.466 | -.629* | -.673* | -.800** |
| MiR203-3p | -.410 | .151 | .477 | -.109 | -.130 | -.515 | -.312 | -.513 |
| MiR203-5p | -.164 | -.127 | -.333 | .127 | .502 | .652** | .329 | .471 |
| MiR493-3p | .164 | .321 | .418 | -.442 | -.665** | -.360 | .018 | -.054 |
| MiR493-5p | -.079 | -.103 | .018 | .212 | .371 | .120 | .079 | .054 |

**Table S4: Spearman correlations between transcript count and behavioral measures.** OF= Open field; WTM= Water T-maze; Bin 1= first 5 minutes of the test. miRNA data were transformed (10-∆∆Ct), so higher values represent higher expression. * *p*<.05 (orange), ** *p*<.01 (red). All significant *p*-values remained significant after FDR correction.

| Gene | Cluster 1 - HP (Standardized factor loading) | Cluster 2 - LP (Standardized factor loading) |
| --- | --- | --- |
| *Slc25a22* | (-) 0.648 | 1.102 |
| *Grin1* | (-) 0.636 | 1.082 |
| *Gabbr1* | (-) 0.632 | 1.075 |
| *Slc17a7* | (-) 0.628 | 1.068 |
| *Nr3c1* | 0.614 | (-) 1.04 |
| *Gabrb1* | 0.604 | (-) 1.027 |
| *Doc2a* | (-) 0.603 | 1.025 |
| *Grik2* | 0.553 | (-) 0.940 |
| *Gls1* | 0.546 | (-) 0.928 |
| *Camk2n2* | (-) 0.544 | 0.926 |
| *Gabrg1* | 0.541 | (-) 0.920 |
| *Grik5* | (-) 0.539 | 0.917 |
| *Slc32a1* | (-) 0.505 | 0.860 |
| *Got2* | (-) 0.491 | 0.835 |
| *Slc12a5* | (-) 0.485 | 0.824 |
| *Slc12a2* | 0.445 | (-) 0.756 |
| *Slc38a7* | (-) 0.441 | 0.751 |
| *Grik1* | 0.439 | (-) 0.747 |
| *Gabrd* | (-) 0.402 | 0.684 |
| *Nr3c2* | 0.351 | (-) 0.597 |

**Table S5**: **Cluster analysis of DEGs in high- and low-performing mice in the T-maze reversal task**. Standardized factor loadings are presented as absolute numbers for ranking convenience, with negative sign indicates that group means were lower in this cluster. The genes Aspa (Aspartoacylase) and Slc25a13 (Mitochondrial Aspartate Glutamate Carrier 2) were excluded from the analysis due to low factor loading (Standardized; <.40) with both clusters.

| Assay | Measure/ Fig # | Statistical test | Effects/Interaction/post-hocs |
| --- | --- | --- | --- |
| Cluster analysis | Cluster difference in RTv | Mann–Whitney | **Z=-3.36, *p*<.001** |
|  | Cluster difference in DEGs |  | **all Zs>3.31, all *p*’s<.001** |
|  | Proportion of C-*Glud1*^+/-^/ Stress mice in cluster 2 | Fisher's exact test (Monte-Carlo simulated) | ***p*=.039; C-*Glud1*^+/-^/ Stress proportion > all other groups** |
| Gene expression in the mPFC at T1 | RT-PCR; ∆∆Ct | Kruskal-Wallis | *Gls1* [H_(2)_=1.02, *p*=.620]  *Grin1* [H_(2)_=2.01, *p*=.377]  *Nr3c1* [H_(2)_=2.92, *p*=.239]  *Vglut1* [H_(2)_=1.80, *p*=.420] |
| miRNA expression in the mPFC at T1 | RT-PCR; ∆∆Ct | Kruskal-Wallis | MiR137-3p [H_(2)_= 2.02, *p*=.380]  MiR137-5p [H_(2)_=0.59, *p*=.756]  MiR203-3p [H_(2)_=3.90, *p*=.143]  **MiR203-5p [H_(2)_=8.56, *p*=.007];** C-*Glud1*^+/-^/ > C-Cre+/ Control: *p*=.007; C-*Glud1*^+/-^/ Stress > C-Cre+/ Stress: *p*=.021.  MiR493-3p [H_(2)_=4.49, *p*=.103]  **MiR493-5p [H_(2)_=9.73, *p*=.002];**C-Cre+/ Stress > C-Cre+/ Control: *p*=.001; C-*Glud1*^+/-^/ Stress > C-Cre+/ Control: *p*=.064.  MiR34a-5p [H_(2)_=0.36, *p*=.846]  **Mean expression of miRNAs [H_(2)_=8.46, *p*=.008];** C-*Glud1*^+/-^/ Stress > C-Cre+/ Control: *p*=.004; C-*Glud1*^+/-^/ Stress > C-Cre+/ Stress: *p*=.039. |
| miRNA expression in the mPFC at T2 | RT-PCR; ∆∆Ct | Kruskal-Wallis | MiR137-3p [H_(2)_= 0.26, *p*=.897]  **MiR137-5p [H_(2)_=10.05, *p*<.001];** C-*Glud1*^+/-^/ Stress > C-Cre+/ Control: *p*=.001;  C-*Glud1*^+/-^/ Stress > C-Cre+/ Stress: *p*=.058.  **MiR203-3p [H_(2)_=10.59, *p*=.001];** C-*Glud1*^+/-^/ Stress < C-Cre+/ Control: *p*=.003;  C-*Glud1*^+/-^/ Stress < C-Cre+/ Stress: *p*=.010.  **MiR203-5p [H_(2)_=6.89, *p*=.024];** C-*Glud1*^+/-^/ Stress < C-Cre+/ Control: *p*=.036; C-*Glud1*^+/-^/ Stress < C-Cre+/ Stress: *p*=.015.  MiR493-3p [H_(2)_=0.38, *p*=.838]  MiR493-5p [H_(2)_=1.31, *p*=.536]  **MiR34a-5p [H_(2)_=9.64, *p*=.001];** C-*Glud1*^+/-^/ Stress < C-Cre+/ Control: *p*=.002;  C-*Glud1*^+/-^/ Stress < C-Cre+/ Stress: *p*=.040.  Mean expression of miRNAs [H_(2)_= 0.10, *p*=.949] |
| miRNA expression in the dCA1 at T2 | RT-PCR; ∆∆Ct | Kruskal-Wallis | MiR137-3p [H_(2)_=3.57, *p*=.173]  **MiR137-5p [H_(2)_=5.87, *p*=.045];** C-*Glud1*^+/-^/ Stress > C-Cre+/ Control: *p*=.047; C-*Glud1*^+/-^/ Stress > C-Cre+/ Stress: *p*=.023.  MiR203-3p [H_(2)_=3.92, *p*=.144]  MiR203-5p [H_(2)_=5.53, *p*=.055]  MiR493-3p [H_(2)_=4.83, *p*=.081]  MiR493-5p [H_(2)_=1.98, *p*=.399]  MiR34a-5p [H_(2)_=2.01, *p*=.387]  **Mean expression of miRNAs [H_(2)_= 7.11, *p*=.019];** C-*Glud1*^+/-^/ Stress > C-Cre+/ Control: *p*=.028; C-*Glud1*^+/-^/ Stress > C-Cre+/ Stress: *p*=.014. |
| miRNA expression in the vCA1 at T2 | RT-PCR; ∆∆Ct | Kruskal-Wallis | MiR137-3p [H_(2)_=0.26, *p*=.890]  MiR137-5p [H_(2)_=1.04, *p*=.641]  MiR203-3p [H_(2)_=0.05, *p*=.981]  MiR203-5p [H_(2)_=0.46, *p*=.817]  MiR493-3p [H_(2)_=1.42, *p*=.540]  MiR493-5p [H_(2)_=3.57, *p*=.173]  MiR34a-5p [H_(2)_=1.18, *p*=.586]  Mean expression of miRNAs [H_(2)_= 2.91, *p*=.244] |

**Table S6: Statistical analysis of mRNA and miRNA expression levels.** T1= Timepoint 1, immediately after stress; T2= Timepoint 2, a month after stress; RTv= reaction time variability.

*miRNA expression in the hippocampus*. Expression of MiR137-3p, MiR137-5p, MiR34a-5p, MiR203-3p, MiR203-5p, MiR493-3p and MiR493-5p was examined at T2 in dCA1 and vCA1 (**Fig S6**). In dCA1, we found no changes in the C-*Glud1*^+/-^/ Stress group relative to C-Cre+/ Controls. Mir-137-5p was upregulated in C-Cre+/ Stress mice compared to C-Cre+/ Control and C-*Glud1*^+/-^/Stress mice. No expression differences were found for other miRNA molecules we examined.

Analysis of the same miRNA molecules in vCA1 (**S6b)** revealed no group differences.

To uncover general patterns of expression changes across miRNAs, we calculated a mean miRNA expression score for each sample. Stress alone induced an increase in mean miRNA expression in dCA1 (C-Cre+/ Stress> C-Cre+/Control). No differences were found in mPFC or in vCA1 (**S7c**).

**Figure S6**: **MiRNA expression in the hippocampus at T2.** (**a**) MiR137-5p was upregulated in dCA1 C-Cre+/ Stress mice. (**b**) No changes in MiRNA expression were found in vCA1. (**c**) A higher mean MiRNA expression score was found in dCA1, but not vCA1 or mPFC, of C-Cre+/ Stress mice.

**References**

1 Lander SS, Linder-Shacham D, Gaisler-Salomon I. Differential effects of social isolation in adolescent and adult mice on behavior and cortical gene expression. *Behavioural Brain Research* 2017; **316**: 245–254.

2 Lander SS, Chakraborti D, Donner R, Provenzano F, Lewandowski N, Mingote SM *et al.* 216. Glutamate Dehydrogenase-Deficient Mice: A Novel Mouse Model of Schizophrenia-Like Phenotypes. *Schizophr Bull* 2017; **43**: S109–S110.

3 Pedersen CS, Sørensen DB, Parachikova AI, Plath N. PCP-induced deficits in murine nest building activity: employment of an ethological rodent behavior to mimic negative-like symptoms of schizophrenia. *Behavioural brain research* 2014; **273**: 63–72.

4 Deacon RMJ. Assessing nest building in mice. *Nat Protoc* 2006; **1**: 1117–1119.

5 Choleris E, Thomas AW, Kavaliers M, Prato FS. A detailed ethological analysis of the mouse open field test: effects of diazepam, chlordiazepoxide and an extremely low frequency pulsed magnetic field. *Neurosci Biobehav Rev* 2001; **25**: 235–260.

6 Archer J. Tests for emotionality in rats and mice: A review. *Anim Behav* 1973; **21**: 205–235.

7 Lee E, Rhim I, Lee JW, Ghim J-W, Lee S, Kim E *et al.* Enhanced Neuronal Activity in the Medial Prefrontal Cortex during Social Approach Behavior. *Journal of Neuroscience* 2016; **36**: 6926–6936.

8 Lander SS, Khan U, Lewandowski N, Chakraborty D, Provenzano FA, Mingote S *et al.* Glutamate Dehydrogenase–Deficient Mice Display Schizophrenia-Like Behavioral Abnormalities and CA1-Specific Hippocampal Dysfunction. *Schizophr Bull* 2019; **45**: 127–137.

9 Ayalon L, Doron R, Weiner I, Joel D. Amelioration of behavioral deficits in a rat model of Huntington’s disease by an excitotoxic lesion to the globus pallidus. *Exp Neurol* 2004; **186**: 46–58.

10 Brooks SP, Janghra N, Higgs G V, Bayram-Weston Z, Heuer A, Jones L *et al.* Selective cognitive impairment in the YAC128 Huntington’s disease mouse. *Brain Res Bull* 2012; **88**: 121–129.

11 Crusio WE, Bertholet J-Y, Schwegler H. No correlations between spatial and non-spatial reference memory in a T-maze task and hippocampal mossy fibre distribution in the mouse. *Behavioural brain research* 1990; **41**: 251–259.

12 Belzung C, Chapillon P, Lalonde R. The effects of the lurcher mutation on object localization, T-maze discrimination, and radial arm maze tasks. *Behav Genet* 2001; **31**: 151–155.

13 Eckner JT, Chandran S, Richardson JK. Investigating the role of feedback and motivation in clinical reaction time assessment. *PM R* 2011; **3**: 1092–1097.

14 Mir P, Trender-Gerhard I, Edwards MJ, Schneider SA, Bhatia KP, Jahanshahi M. Motivation and movement: the effect of monetary incentive on performance speed. *Exp Brain Res* 2011; **209**: 551–559.

15 Herremans SC, Vanderhasselt M-A, De Raedt R, Baeken C. Reduced Intra-individual Reaction Time Variability During a Go–NoGo Task in Detoxified Alcohol-Dependent Patients After One Right-Sided Dorsolateral Prefrontal HF-rTMS Session. *Alcohol and Alcoholism* 2013; **48**: 552–557.

16 Shammi P, Bosman E, Stuss DT. Aging and Variability in Performance. *Aging, Neuropsychology, and Cognition* 1998; **5**: 1–13.

17 Zaidan H, Leshem M, Gaisler-Salomon I. Prereproductive Stress to Female Rats Alters Corticotropin Releasing Factor Type 1 Expression in Ova and Behavior and Brain Corticotropin Releasing Factor Type 1 Expression in Offspring. *Biol Psychiatry* 2013; **74**: 680–687.

18 Zaidan H, Galiani D, Gaisler-Salomon I. Pre-reproductive stress in adolescent female rats alters oocyte microRNA expression and offspring phenotypes: pharmacological interventions and putative mechanisms. *Transl Psychiatry* 2021; **11**: 113.

19 Rozen S, Skaletsky H. Primer3 on the WWW for General Users and for Biologist Programmers. In: *Bioinformatics Methods and Protocols*. Humana Press: New Jersey, 2000, pp 365–386.

20 Pfaffl MW, Tichopad A, Prgomet C, Neuvians TP. Determination of stable housekeeping genes, differentially regulated target genes and sample integrity: BestKeeper – Excel-based tool using pair-wise correlations. *Biotechnol Lett* 2004; **26**: 509–515.

21 Livak KJ, Schmittgen TD. Analysis of relative gene expression data using real-time quantitative PCR and the 2(-Delta Delta C(T)) Method. *Methods* 2001; **25**: 402–408.

22 Szklarczyk D, Gable AL, Lyon D, Junge A, Wyder S, Huerta-Cepas J *et al.* STRING v11: protein-protein association networks with increased coverage, supporting functional discovery in genome-wide experimental datasets. *Nucleic Acids Res* 2019; **47**: D607–D613.

23 Paraskevopoulou MD, Georgakilas G, Kostoulas N, Vlachos IS, Vergoulis T, Reczko M *et al.* DIANA-microT web server v5.0: service integration into miRNA functional analysis workflows. *Nucleic Acids Res* 2013; **41**: W169–W173.

24 McGeary SE, Lin KS, Shi CY, Pham TM, Bisaria N, Kelley GM *et al.* The biochemical basis of microRNA targeting efficacy. *Science* 2019; **366**. doi:10.1126/science.aav1741.
